# Repair of Iatrogenic Furcal Perforation With Mineral Trioxide Aggregate: A Case Report

**DOI:** 10.7759/cureus.62035

**Published:** 2024-06-09

**Authors:** Shreeya Panchal, Manoj Chandak, Jay Bhopatkar, Paridhi Agrawal, Akanksha Gupta, Neha Pankey

**Affiliations:** 1 Department of Conservative Dentistry and Endodontics, Sharad Pawar Dental College and Hospital, Datta Meghe Institute of Higher Education and Research, Wardha, IND; 2 Department of Pediatric and Preventive Dentistry, Sharad Pawar Dental College and Hospital, Datta Meghe Institute of Higher Education and Research, Wardha, IND

**Keywords:** conservative approach, furcal perforation repair, perforation repair, iatrogenic perforation, mineral trioxide aggregate [mta]

## Abstract

In endodontic and restorative procedures, an accidental perforation of the pulp chamber floor or roots presents a considerable risk, potentially leading to persistent inflammatory responses and ultimately tooth loss. Accidental root canal perforations are primary complications encountered by clinicians, requiring either surgical or non-surgical intervention, depending on the severity of the perforation. Over the years, various materials have been utilized for the treatment of such complications, but mineral trioxide aggregate (MTA) stands out prominently due to its exceptional biocompatibility, remarkable sealing capacity, and potent antibacterial properties. The unique ability of MTA to set in the presence of moisture facilitates the formation of a robust seal, thereby making it highly effective in managing root perforations and fostering tissue regeneration within the affected area. Its versatility and effectiveness have made MTA a cornerstone material in modern endodontic therapy, offering clinicians a reliable solution for enhancing the long-term prognosis of teeth affected by perforations.

## Introduction

Inadvertent perforation of the pulp chamber floor or roots is a significant risk associated with endodontic and restorative procedures. Such a perforation can happen during nonsurgical root canal therapy or as part of the setup for a number of other restorative operations [1]. Consequently, there is a persistent inflammatory response in the periodontium, which is characterized by the formation of granulation tissue. This response may result in tooth loss or irreversible attachment loss [2]. Root canal perforations occurring accidentally represent one of the primary complications encountered during endodontic procedures [3]. Depending on the unique circumstances of each case, either surgical or non-surgical procedures are employed to treat these perforations. The outcome is generally better if the issue is accurately detected or treated with a substance that has the appropriate sealing capacity and biocompatibility. However, the prognosis may be challenging if therapy entails a lesion occurring at the level of the radicular furcation [4].

An array of materials, including amalgam, calcium hydroxide, glass ionomer, composite resin, zinc oxide-eugenol, and resin-modified glass ionomer, have been used to address perforations. Besides being nontoxic, non-absorbable, radiopaque, bacteriostatic, or antibacterial, the ideal material for managing radicular perforations should also act as a sealant to hinder microleakage from the perforated area [5]. All of these traits are present in mineral trioxide aggregate (MTA), which is being used with success in root-end surgery, direct pulpal covering root resorption and specification, and the restoration for furcal and radicular perforations. Its biocompatibility, low inflammatory induction, solubility, ability to form a seal between the pulpal chamber and periodontal tissues, and ability to heal all contribute to its potential for handling all these concerns [6].

The final feature can be ascribed to the antibacterial characteristics and elevated pH (12.5) of MTA, which foster the development of the cementum and bone, thereby permitting the regeneration of the periodontal ligament surrounding the site of injury [7]. The ability of MTA to set in the presence of moisture makes it advantageous for promoting a strong seal when utilized in root perforations [8].

The goal of treating a furcal perforation is to block the artificial link between the endodontic space and the periradicular tissue to avoid alveolar bone loss and injury to the ligament that supports the periodontal tissue [9].

## Case presentation

**Figure 1:**
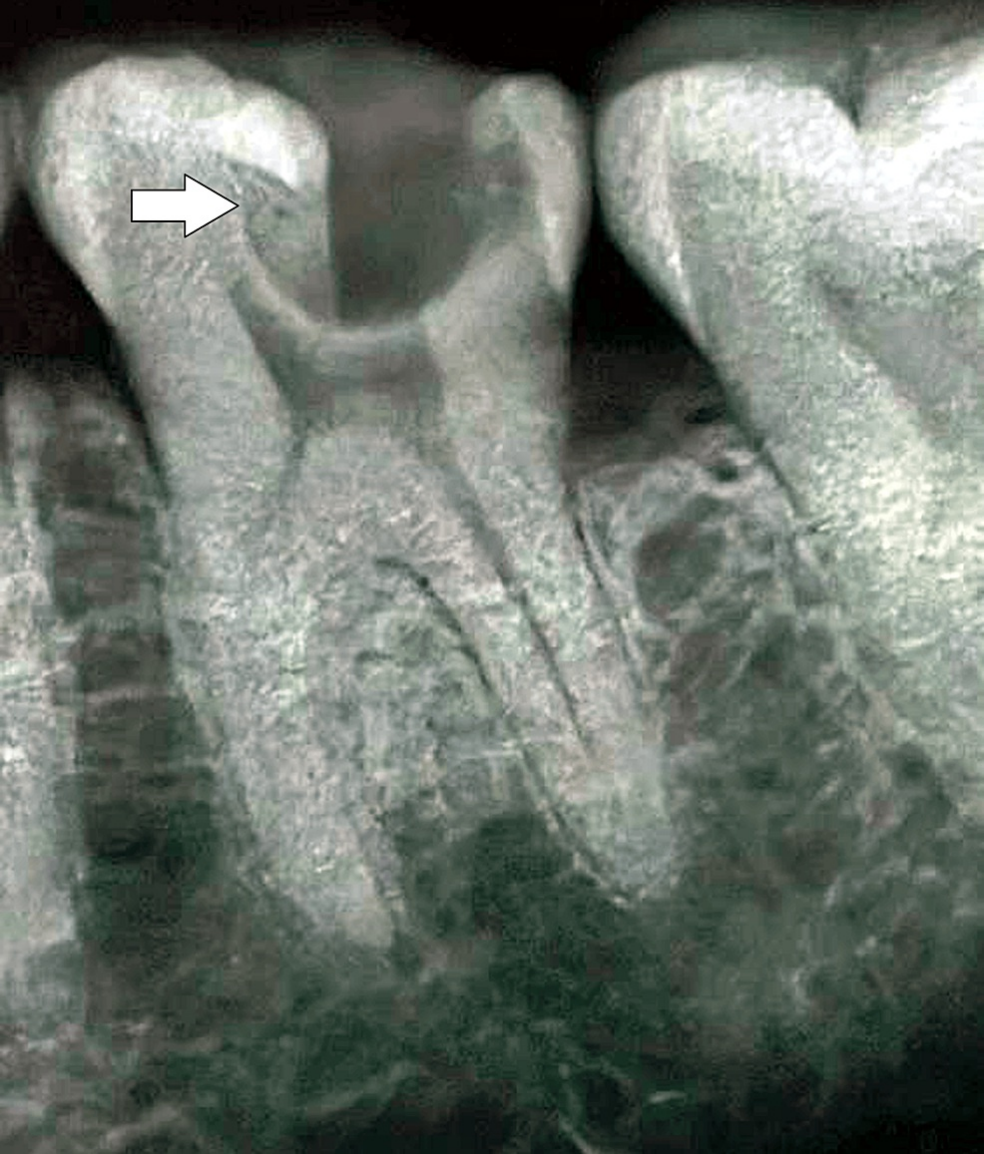
Diagnostic radiograph showing deep occlusal caries in tooth 46 Courtesy: Shreeya Panchal

A 27-year-old male patient from Wardha presented with pain to the endodontics and conservative dentistry department at the Sharad Pawar Dental College and Hospital in Sawangi, Wardha. He has been experiencing pain in the lower right back region of the jaw for the last two months. Initially, in the past couple of weeks, he has observed an increase in pain from the formerly minor and intermittent condition. The patient reports sensitivity to hot or chilled food. The pain comes on suddenly and gets worse, especially when lying down at night. There is no significant past medical history or relevant dental history. Additionally, there are no notable habits associated with the patient's condition. During the clinical examination, deep occlusal caries were observed on tooth 46 (Figure 1), accompanied by tenderness on percussion.

**Figure 2:**
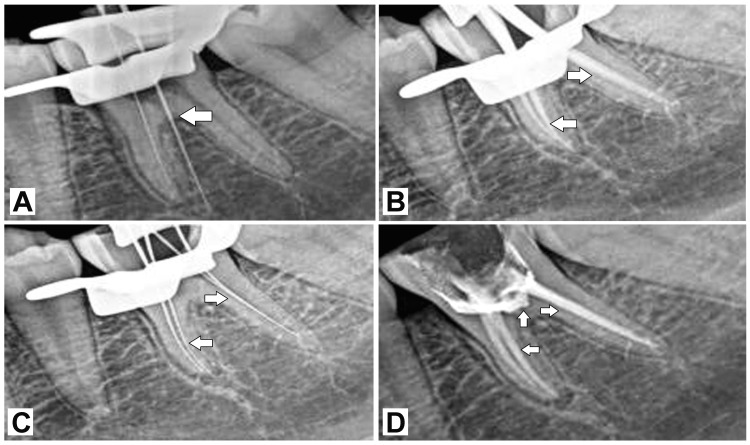
Radiographic insights of perforation and treatment insights: (A) radiograph showing perforation in furcation region with 46; (B) radiograph showing master cone selection with 46; (C) re-identified canals with 46; (D) obturated canals with gutta percha with 46 and perforation sealed with MTA with 46 Courtesy: Shreeya Panchal

Additionally, stains and calculus were present. Based on these findings, the provisional diagnosis is symptomatic irreversible pulpitis with symptomatic apical periodontitis concerning tooth 46. The examination further supported the diagnosis, showing radiolucency of the carious lesion involving the pulp, as well as mild widening of the periodontal ligament space associated with tooth 46. The final diagnosis made was symptomatic irreversible pulpitis with symptomatic apical periodontitis for tooth 46. The treatment plan for this condition comprises several phases: urgent, control, re-evaluation, definitive, and maintenance. The urgent phase was deemed to be not necessary. In the control phase, root canal opening and biomechanical preparation were performed on tooth 46, along with oral prophylaxis. Subsequently, during the procedure, an accidental perforation occurred in the furcation region of tooth 46, which was confirmed radiographically (Figure 2A).

Hemostasis of the perforated area was achieved using 1:100,000 adrenaline containing 2% local anesthesia (Septocaine, Septodont, Saint-Maur-des-Fossés, France). The procedure was continued by re-identifying the canals and determining the working length radiographically. Following this, the canals were cleaned and shaped. Rotary files were utilized to clean and shape the mesio-buccal and mesio-lingual till W2 (20-6%, Woodpecker 2019 Endoplus, Woodpecker Medical Instruments, Guilin, China) and distal till W3 (25-6%, Woodpecker 2019 Endoplus, Woodpecker Medical Instruments, Guilin, China) followed by irrigation with 5% sodium hypochlorite (Parcan, Septodont, Saint-Maur-des-Fossés, France) and a final rinse with 2% chlorhexidine (Chloro-Hx, Maarc, Vasai-Virar, India), Then, an appropriate size of master gutta-percha cone was selected and confirmed radiographically (Figure 2B).

Subsequently, 10K file (Mani, Utsunomiya, Japan) was placed into the canal and the position of the canals was confirmed. This was done before blocking the canals (Figure 2C). Afterward, gutta-percha points (Dia-Pro ISO.06, DiaDent, Cheongju-si, South Korea) and resin-based sealer (Dia-Pro Sealer, DiaDent, Cheongju-si, South Korea) were used for obturation, and the perforation was sealed with mineral trioxide aggregate (MTA) paste (ProRoot MTA, Dentsply Sirona, Charlotte, NC, USA) (Figure 2D). When the patient was called after 24 hours for assessment, he did not report any pain or symptoms.

**Figure 3:**
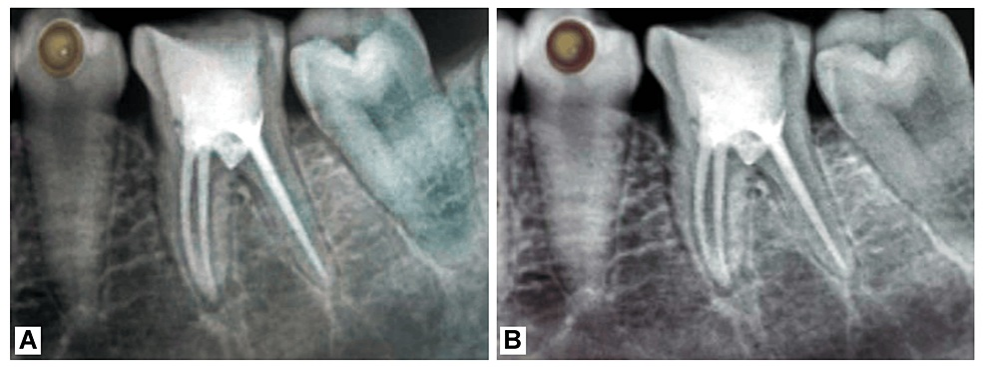
Post-operative radiographs: (A) immediate post-operative radiograph of 46; (B) six-month follow-up post-operative radiograph of 46 Courtesy: Shreeya Panchal

At this visit, the temporary sealing material was removed, and the hardness of the MTA was tested before permanent restoration with microhybird composite resin (Spectrum, Dentsply Sirona, Charlotte, NC, USA) (Figure 3A).

Errors occurred during the procedure, including improper pre-operative assessment of root canal anatomy and failure to estimate the pulpal depth using pre-operative radiographs. Additionally, there was a mistake in performing the complete access opening using only a round diamond bur, without switching to a safe end bur after the initial bur drop. A six-month follow-up radiograph showed no sign of any periapical pathology or bone loss with no furcal pathology (Figure 3B).

## Discussion

During root canal treatment or post-preparation, furcal perforation can emerge as an unwanted complication. Likewise, the removal of an infected tissue in patients with caries affecting the pulpal chamber also carries the risk of perforation [10]. In these scenarios, swift intervention is crucial to ensure a positive prognosis. Immediate repair of the perforation is a primary goal to minimize bacterial contamination and inflammatory processes. This helps enhance post-treatment outcomes within the defect area. Elements like location, size, timing of contamination, and the material employed for repair significantly influence the prognosis of perforations [11]. In the present scenario, the issue was quickly resolved by employing MTA. Furcal perforation is an undesired issue that can arise during root canal therapy or following preparation [12]. Similarly, in patients whose caries affect the pulpal chamber, there is a danger of perforation during the removal of the infected tissue. In any case, quick resolution is essential because it helps ensure a favorable prognosis right away. One of the primary objectives in managing perforations is the immediate repair to minimize the risk of bacterial contamination and inflammatory processes in the problem area, leading to better results after treatment [13]. The location, size, and duration of the lesion's contamination, as well as the type of material employed for repair, all affect the prognosis of perforations [14]. In this instance, the issue was quickly resolved with the use of MTA. 

**Figure 4:**
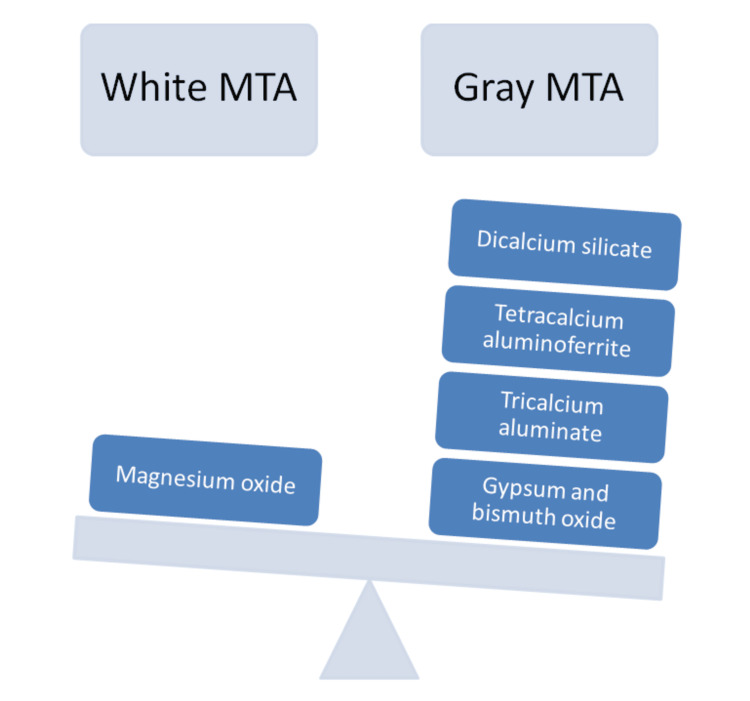
Difference between white and gray MTA Courtesy: Shreeya Panchal

MTA functions similarly in terms of furcal sealing and antibacterial efficacy in both the gray and white forms [15] (Figure 4).

While the white variant is linked to the formation of keratinocytes and cementoblasts, the gray form exhibits more favorable behavior for odontoblast development in vitro. This is necessary for fixing furcation. Because MTA delays the setting time and granular uniformity, it is challenging to work with [16]. When employing this kind of material, it is best to avoid contaminating the blood because this can lower the MTA's retention ability. In this scenario, the closure of the lesions was noticeable, accompanied by some extrusion of the material. Some authors propose that to avoid overfilling or underfilling, it may be beneficial to apply a resorbable collagen matrix prior to placing the MTA; however, the decision to use a matrix depends on the size of the lesion. Materials for molar tooth restoration and furcal perforation repair must adapt to occlusal stresses [17]. Some studies have reported a maximum bite force of 640 N across all teeth, with one tooth registering a force of 265 N in comparison to amalgam, Super-EBA, and intermediate restorative material (IRM). MTA showed the lowest compressive strength (40 MPa) after 24 hours. However, after 21 days, its strength improved to 67 MPa [18]. Due to its limited compressive strength, MTA shouldn't be used in functioning areas [19]. Despite the fact that MTA has been recommended for use in a number of endodontic treatments, research on its effectiveness in repairing furcal perforations has demonstrated its high quality [20]. 

## Conclusions

Swift intervention is vital for managing furcal perforations and pulpal chamber infections during root canal treatment to ensure a positive prognosis. Immediate repair with materials like MTA minimizes bacterial contamination and inflammation, enhancing post-treatment outcomes. The location, size, timing of contamination, and repair material significantly affect prognosis. MTA, available in gray and white forms, exhibits similar furcal sealing and antibacterial efficacy, with the gray variant favoring odontoblast development. However, MTA's delayed setting time and granular consistency present challenges during application, emphasizing the importance of avoiding blood contamination to maintain its retention ability. While a resorbable collagen matrix may aid in optimizing the application, its necessity depends on lesion size. Materials for furcal perforation repair must withstand occlusal stresses, considering MTA's limited initial compressive strength though it improves over time. Despite its limitations, research demonstrates MTA's effectiveness in repairing furcal perforations, highlighting its role in endodontic treatments while cautioning against its use in functioning areas.
